# Monkeypox Vaccine Hesitancy in French Men Having Sex with Men with PrEP or Living with HIV in France

**DOI:** 10.3390/vaccines10101629

**Published:** 2022-09-28

**Authors:** David Zucman, Erwan Fourn, Pauline Touche, Catherine Majerholc, Alexandre Vallée

**Affiliations:** 1 Department of Internal Medicine, Réseau Ville Hôpital Val de Seine, Foch Hospital, 92150 Suresnes, France; 2 Department Epidemiology-Data-Biostatistics, Delegation of Clinical Research and Innovation (DRCI), Foch Hospital, 92150 Suresnes, France

**Keywords:** monkeypox virus, epidemic, vaccine, vaccine hesitancy, PrEP, HIV, gay, men

## Abstract

People with pre-exposure prophylaxis (PrEP) or living with HIV are a high-risk population for monkeypox virus (MPXV) infection. It is important to achieve high MPXV vaccination coverage rates in this group. This project used self-reporting to assess vaccine hesitancy for the smallpox vaccine and acceptance among men having sex with men with PrEP or living with HIV. In total, 52 (33.6%) participants among the 155 declared their hesitancy to be vaccinated against MPXV. Moreover, 20.7% patients with PrEP declared a hesitant attitude towards the smallpox vaccine compared to 40.2% of the HIV patients, *p* = 0.013. This difference remained not significant after adjustment for age (*p* = 0.119) and after adjustment for both age and number of different sexual partners (*p* = 0.406). Among PrEP people, those who expressed concerns about people getting more vaccines than needed (*p* = 0.012) were less likely to accept vaccination, whereas an increased number of different sexual partners during the previous month was significantly associated with acceptance of vaccination (*p* = 0.034). Among HIV people, those who expressed concerns about being infected by MPXV (*p* < 0.001), those who expressed that the smallpox vaccine should be compulsory for people at risk (*p* < 0.001) and those with an increased the number of different sexual partners the previous month (*p* = 0.018) were significantly associated with higher acceptance of MPXV vaccination. Our results suggest that vaccine strategy would be efficient in France with a communication strategy emphasizing the benefits of vaccination and the potential MPXV risk infection for health in PrEP and HIV people. Other preventive actions should be implemented, including reduction in sexual partners.

## 1. Introduction

The emergence of the monkeypox virus (MPXV) outbreak in 2022 has posed another global health threat [1]. To date, more than 58,000 laboratory-confirmed cases and 18 deaths have been reported to the World Health Organization (WHO) from 103 countries/territories/areas in all six WHO Regions [2]. As of 15 September 2022, the cumulative confirmed case numbers in France was 3898 [3]. Due to the rapid increase in monkeypox cases, the WHO declared the escalating global monkeypox outbreak a Public Health Emergency of International Concern on 23 July 2022.

Like previous infectious diseases [4], monkeypox is a contagious disease which requires physical or sexual contact with someone infected with the virus [5]. Although the risk to the general public was considered low previously, the WHO is responding to this outbreak as a high priority to avoid further spread [6]. In addition to those who have close contact with patients with monkeypox or contact with an infected animal, immunocompromised patients may also be at risk of acquiring monkeypox [7]. Several studies have reported the development of monkeypox in patients with HIV during this outbreak [8,9,10,11,12]. The main reason could be that these HIV cases carry the same risk as men who have sex with men [13]. Moreover, a recent analysis showed numerous preliminary risk factors, such as being a young man, having sex with other men (MSM), engaging in risky behaviors and activities such as condomless sex, PrEP (pre-exposure prophylaxis) and HIV positivity [13,14]. To this date, there is no standard-of-care therapy for monkeypox except supportive care [15]. Although smallpox antivirals with poxvirus activity, such as cidofovir, brincidofovir and tecovirimat, are active against the monkeypox virus, these antivirals would most likely be reserved for the treatment of severe cases or immunocompromised persons [15]. In addition, smallpox vaccines have been proposed to the specific risk population to counteract the MPXV outbreak: ACAM2000 orthopoxvirus and JYNNEOS vaccines [16,17]. In France, the Health High Authority (Haute Autorité de Santé, HAS) recommends vaccination of people exposed to the virus, MSM and people in prostitution. People living with HIV, MSM and people with PreP may need priority status for the smallpox vaccine. Thus, it is essential to understand the possible reasons for refusing the smallpox vaccine to better respond to their worries or hesitancies. This study focuses on smallpox vaccine hesitancy and its determinants in a French population of people living with HIV or with PreP.

## 2. Materials and Methods

The study was conducted in July and August 2022 among MSM persons living with HIV followed up for this diagnosis in Foch hospital and among MSM with men with PrEP. In the Foch hospital, Suresnes, France, 361 patients (252 living with HIV and 109 with PrEP) were followed up and gave their e-mail addresses. Mean duration of HIV infection is 18 years; 98% of those infected are receiving antiretroviral therapy and in 95% of them, HIV viral load is undetectable on treatment. These patients have twice yearly outpatient visits. All patients were contacted to participate in this study. Participants with no idea of either vaccine acceptance or vaccine hesitancy were excluded from analyses. All the patients included declared that they are free of MPXV.

An anonymous online survey was developed based on past research involving attitudes and behavior about vaccination [18,19,20]. The survey assessed (Supplementary file):

- Demographic characteristics of participants;
- General attitudes and perception to vaccines;
- MPXV—personal opinions;
- Personal views—MPXV and vaccines;
- Personal experience with MPXV.

Two e-mail reminders were sent out to obtain the maximum number of responses.

According to the WHO Strategic Advisory Group of Experts on Immunization, vaccine hesitancy was defined as “delay in acceptance or refusal of vaccine despite availability of vaccination services” [21].

The study was approved by the Foch IRB: IRB00012437 (approval number: 22-07-05) on 22 July 2022. Non-opposed consent was obtained for all participants.

### Statistical Analysis

We computed descriptive statistics to describe the demographic characteristics of the study participants. The analyses were stratified among two groups, HIV people and PrEP people. Continuous variables were described as mean and SD (standard deviation) and categorical variables were described as number and percentage (%). Fisher’s Exact test or Pearson’s chi-square and Mann–Whitney tests were used to identify significant differences between participants who would accept the smallpox vaccine or be hesitant. Responses were compared by dichotomizing the variable as a positive (yes) or hesitant attitude (no or not for the moment) towards a smallpox vaccine indicating the extent of vaccine hesitancy. Covariates were continuous for the number of sexual partners and categorical for the other questions. Multiple backward logistic regression was performed to identify the predictors of smallpox vaccine acceptance in the two groups of PrEP and HIV patients. Independent variables were defined by the multiple regression. AUC (area under the ROC curve) was performed to investigate the performance of the models. Statistical significance was established at an alpha of *p* < 0.05. Data were analyzed using SAS software (version 9.4; SAS Institute, Carry, NC, USA).

## 3. Results

The survey was completed by 102 of the 252 patients living with HIV (response rate = 40.5%) and by 53 of the 109 patients with PrEP (response rate = 48.6%).

The overall sample was 100% male and 80.64% of participants were aged between 30 and 59 years (Table 1).

In total, 52 (33.6%) participants among the 155 declared their hesitancy to be vaccinated against MPXV.

Of the participants, 6 (3.9%) declared that they have been directly confronted with MPXV and 23 (14.8%) through a person known to them. None of the participants declared that they had been infected by MPXV infection.

Hesitancy towards vaccines in general was very low, only 27 (17.4%) of the study population thought that French people receive too many vaccines, but 96 (61.9%) declared that they had agreed to get vaccinated despite doubts about its effectiveness. A total of 42 (27.1%) of the participants had concerns about the smallpox vaccine, but 85 (54.8%) felt at risk of being infected with the MPXV.

HIV patients were older than PrEP patients (*p* < 0.001), but showed less STI infections (16% vs. 34%, *p* = 0.014), and fewer different sexual partners in the last month (2.9 vs. 5.3, *p* = 0.006) and in the last 3 months (5.5 vs. 11.8, *p* = 0.002) (Table 1).

In total, 11 (20.7%) patients with PrEP declared a hesitant attitude towards the smallpox vaccine compared to 41 (40.2%) of the HIV patients, *p* = 0.013 (Table 2).

**Figure 1:**
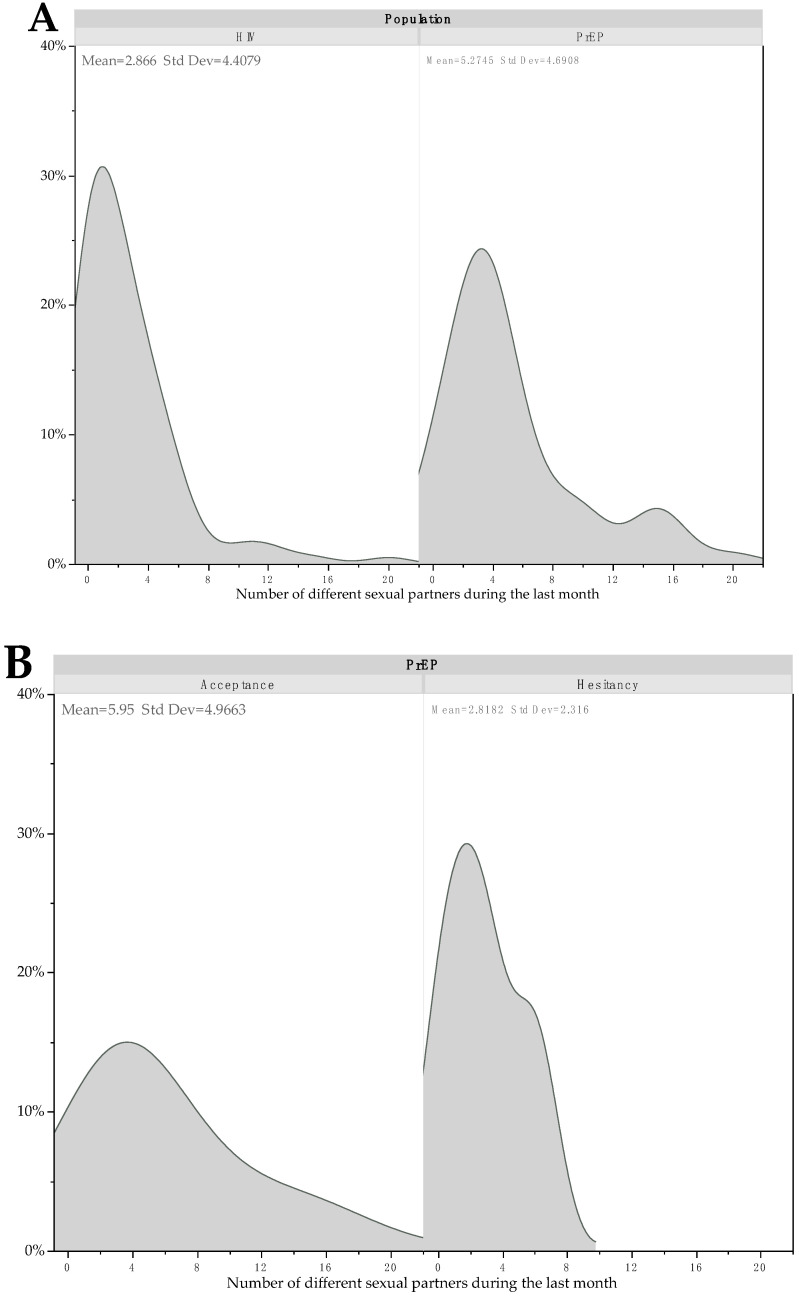

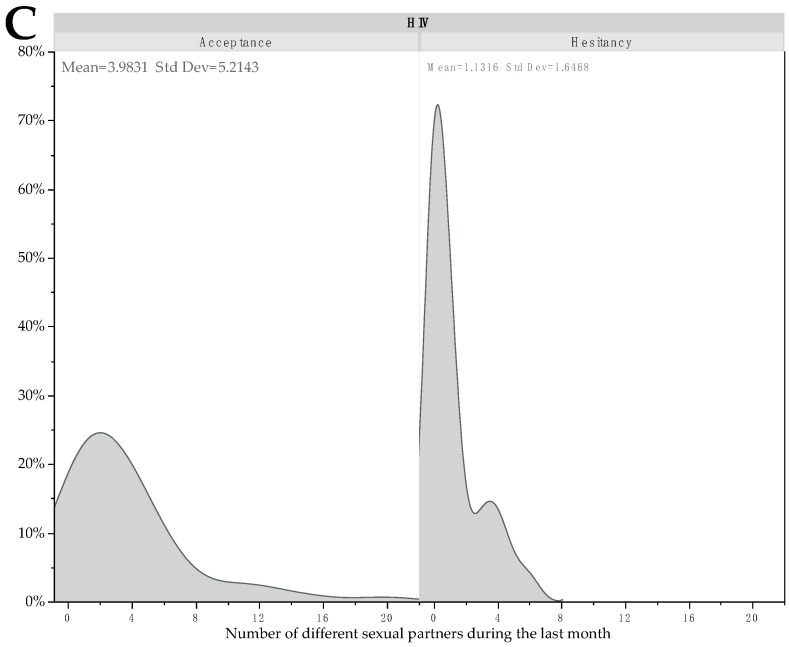
Kernel density plots for number of different sexual partners the last month according to HIV and PrEP status (**A**), and hesitancy or not in PrEP patients (**B**) and in HIV patients (**C**).

This difference remained not significant after adjustment for age (*p* = 0.119) and after adjustment for both age and number of different sexual partners during the last month (*p* = 0.406) (Figure 1).

Among PrEP patients, those who declared being hesitant felt less at risk of being infected by MPXV (45.5% vs. 83.3%, *p* = 0.032). Fewer of them felt that the smallpox vaccine was important in reducing the spread of the outbreak (45.5% vs. 95.2%, *p* < 0.001), but a higher number thought that people received more vaccines than they need (45.5% vs. 7.1%, *p* < 0.001) and more were concerned about serious side effects from the PXV vaccine (54.6% vs. 26.2%, *p* = 0.049). Moreover, PrEP patients with a positive attitude towards vaccination presented the highest number of different sexual partners during the last month (6.0 vs. 2.8, *p* = 0.019), but not during the last three months (*p* = 0.149) (Table 2).

In multiple logistic regression, two different items (items 14 and 25 of Table 2) were predictive of MPXV vaccination hesitancy. Item 14, which expressed concerns about people getting more vaccines than they needed (*p* = 0.012), and Item 25, for the number of different sexual partners in the last month (*p* = 0.034), were significantly associated with acceptance of vaccination (Table 3).

**Figure 2:**
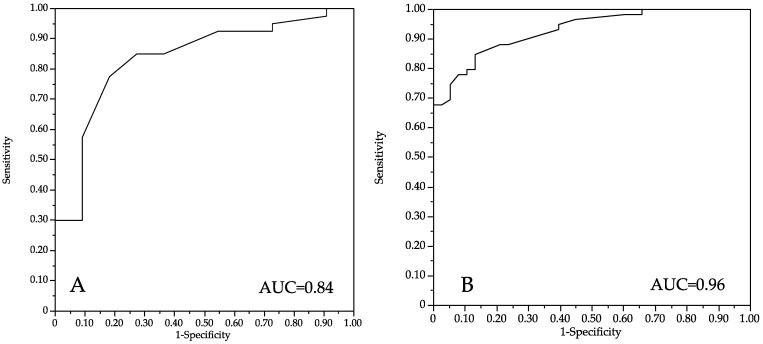
ROC AUC for performance of the multiple regression models in PrEP patients (**A**) and in HIV patients (**B**).

The performance expressed by the AUC was 0.84 (Figure 2).

Among HIV patients, those who declared a hesitant attitude felt less at risk of being infected by MPXV (7.5% vs. 68.9%, *p* < 0.001), fewer thought that vaccine were important to stay healthy (87.5% vs. 100%, *p* = 0.005), fewer of them thought that the smallpox vaccine was important in reducing the spread of the outbreak (77.5% vs. 96.7%, *p* = 0.009), fewer thought that the smallpox vaccine should be compulsory for people at risk (30.0% vs. 72.1%, *p* < 0.001) and fewer thought that MPXV vaccination is important for patient with chronic disease (40.5% vs. 91.2%, *p* < 0.001), but more thought that people received more vaccines than they need (33.3% vs. 9.8%, *p* = 0.003). Moreover, HIV patients with a positive attitude towards vaccination presented the highest number of different sexual partners during the last month (3.9 vs. 1.6, *p* = 0.002) and during the last three months (7.9 vs. 1.8, *p* = 0.001) (Table 2).

In multiple logistic regression, three different items (items 10, 17 and 25 of Table 2) were predictive of MPXV vaccination hesitancy. Item 10, which expressed concerns about being infected by MPXV (*p* < 0.001), Item 17, which expressed that the smallpox vaccine should be compulsory for people at risk (*p* < 0.001), and Item 25, for the number of different sexual partners in the last month (*p* = 0.018), were significantly associated with acceptance of vaccination (Table 3). The performance expressed by the AUC was 0.96 (Figure 2).

## 4. Discussion

Since March 2020, the world has been faced with the burden of the COVID-19 pandemic, which is now coupled with MPXV for a specific population, that is especially MSM people [12,22]. In the United States, most MPXV cases have occurred among MSM who have a higher prevalence of HIV and STIs than the general population [22]. MPXV infection most often begins with a fever, which is frequently high and accompanied by headaches, body aches and asthenia. After about 2 days, a blistering rash appears, made up of fluid-filled blisters that progress to drying out, scab formation and then scarring. The vesicles tend to be concentrated on the face, the palms of the hands and the soles of the feet, but also in the mouth and the genital area. The incubation of the disease can range from 5 to 21 days. The disease most often heals spontaneously, after 2 to 3 weeks. MPXV can be transmitted by direct contact with skin lesions or mucous membranes of a sick person, as well as by droplets (saliva, sneezing, sputters, etc.). Cases can also become contaminated through contact with the patient’s environment [5]. A high number of reports on transmission have highlighted unprotected intercourse with numerous sex partners [5,23]. Thus, the contact with breached anogenital mucosal membranes may be an unrecognized transmission mode of MPXV among MSM people [24]. This pandemic has caused problems for people, at both socioeconomic and health levels. In a recent report of 528 monkeypox infections (27 April to 24 June 2022) in 16 countries, the majority (98%) were MSM men; with the median age of 38 years [10]. Most (95%) presented with a rash, 73% had lesions in the anogenital areas and 41% with lesions in the mucosa. The systemic features were fever (62%), lymphadenopathy (56%), fatigue (41%), myalgia (31%) and headaches (27%). Thus, the new MPXV pandemic could potentially present a detrimental effect on specific populations, such as PrEP people and HIV patients regarding worry and anxiety [25].

Moreover, we are worried that epidemiological investigations have shown no substantial travel associations of the European cases and the monkeypox-endemic areas in Africa [26]. This could be the result of an undetected spread in Europe for a while, with human to human transmission, which occurred by close physical contact with infected asymptomatic or symptomatic adults [27]. MPXV vaccination was discontinued worldwide in the 1980s, the increased number of men in the young age group could reflect a loss of cross-protective immunity to MPXV [28]. The majority of the European cases were men who have sex with men [29]. Knowledge of monkeypox risk appears to be low at present in the gay community. Seeking vaccination is associated with higher worry levels [30]. Moreover, healthy behavior is generally associated with vaccine acceptance [31].

In our study, 52 (33.6%) participants among the 155 declared their hesitancy to be vaccinated against MPXV. Furthermore, 11 (20.7%) patients with PrEP declared a hesitant attitude towards the smallpox vaccine compared to 41 (40.2%) of the HIV patients. This indicates that, in our sample, around than 4 of 10 people living with HIV and 2 of 10 people with PrEP were vaccine-hesitant despite self-perception of elevated risk of exposure to COVID-19 infection. To this date, very few studies have focused on the interest of the hesitancy towards the smallpox vaccine. A previous study performed in Saudi Arabia showed that 60.4% of the participants indicated their higher worry about the MPXV outbreak but only 50.6% agreed with MPXV vaccination [31]. This result could be compared to the 75.5% of PrEP participants and 44.6% HIV patients who felt at risk of being infected by MPXV (item 10), i.e., 54.8% of the study population and 79.3% for PrEP people and 59.8% for HIV people who agreed to be vaccinated (i.e., 66.4% of the overall study population).

Nevertheless, we observed that 33.6% of the participants declared being hesitant to MPXV vaccination. We found that MPXV vaccination acceptance behavior was strongly associated with certain characteristics of the participants: number of different sexual partners during the last month for both the two groups (Item 25), fear about MPXV infection Item 10) and willingness to make COVID-19 vaccination mandatory (Item 17). In contrast, among PrEP participants, general doubts about vaccination (Item 14) were a factor associated with hesitancy to be vaccinated among PrEP people. Thus, immediate long-term public and specific messages focused on the benefits of the vaccination are needed [32]. General vaccination against MPXV in the current stage of the disease is a challenging decision for healthcare policy authorities. Public perception regarding the acceptance of this type of decision if taken for specific groups, e.g., PrEP people and HIV patients, needs to be investigated considering the COVID-19 vaccine hesitancy the public went through. These preventive messages could be the reduction in sexual partners in association with vaccination and specific messages on the benefits of MPXV vaccination. However, the optimal strategy to offer vaccine like PrEP needs to be considered. Thus, like the French health authorities, it is necessary to recommend a vaccine to MSM who self-identify as having multiple partners or to those who are already under treatment for other infectious sexually transmitted diseases, such as HIV, syphilis and gonorrhea. However, an overall low level of knowledge of MPXV has been observed among physicians in Kuwait. A previous study has shown that only 50.1% of medical doctors were confident in a diagnosis based on a diagnostic test, 47.5% to manage MPXV and only 32.2% to diagnose MPXV clinically [33]. Thus, the management of this outbreak should consider the formation, education, and training of physicians. Similar results were observed among Italian physicians [34] and healthcare workers in Jordan [35]. The smallpox vaccine against the eradicated MPXV virus has been shown to have an 85% cross protective effect [36]. However, it remains unclear how long the protection lasts for this new MPXV pandemic. An 85% protective efficacy was obtained with the first-generation vaccinia virus vaccines against transmission through droplet spread [36].To this date, we do not know if immunization with a smallpox vaccine will provide a real effective protective immunity (more than 85%) against sexually transmitted MPXV. Therefore, the use of a vaccine against MPXV must be investigated in randomized and controlled trials.

### Limitations Section

Limitations of this study include data collection at a single medical hospital, which may impact generalizability. Our study was internet-based; therefore, we could not eliminate a selectivity bias. Our study was a cross-sectional study, so no causality can be established. The questionnaire was designed to be simple and easy-to-answer, so we could not evaluate daily habits. The small sample size and potential selection, measurements and social desirability should be considered as the main bias in our study and limit the generalization of our results. Moreover, the binary responses, such as yes/no, limit the interpretation of all acceptance points of view of the participants. The French ethical aspects of our anonymous questionnaire did not allow us to question the participants about their specific socio-economic and educational status (more than we reported). This did not allow us to compare our results to literature focused on these topics. Respondents could also have been predominantly influenced by exposure to smallpox vaccine-related topics in the media, no information for time of exposure to media or other media information and propaganda from public institutions for the spread of smallpox vaccines were collected in the online questionnaire.

## 5. Conclusions

Most of this cohort had positive attitudes regarding MPXV vaccination, comparable to the few prior studies. Despite limitations, this study sheds light on smallpox vaccine hesitancy among participants living with HIV and PrEP. Our results suggest that a vaccine strategy should be implemented in France with a communication strategy emphasizing the collective benefits of herd immunity in the population living with HIV and in the population of PrEP people and focusing on MPXV risk for health. The management of this outbreak should consider a public health education of the population at risk by specific media information or education by physicians to ensure the knowledge of this outbreak and its health-associated risk.

## Figures and Tables

**Table 1:** Characteristics of the study population.

	PrEP Patients		HIV Patients		
	*n* = 53	%/SD	*n* = 102	%/SD	*p* Value
**Item 1—Do you want to get vaccinated against MPXV? (yes)**	**42**	**79.25%**	**61**	**59.80%**	**0.013**
Item 2—Age					**<0.001**
*18–29 years*	5	9.43%	4	3.92%	
*30–39 years*	24	45.28%	13	12.75%	
*40–49 years*	14	26.42%	29	28.43%	
*50–59 years*	10	18.87%	35	34.31%	
More than 60 years old	0	0.00%	21	20.59%	
Item 3—Area of Origin					0.839
*Africa*	3	5.66%	8	7.92%	
*America*	2	3.77%	3	2.97%	
*Asia/Oceania*	4	7.55%	4	3.96%	
*Europe*	43	81.13%	85	84.16%	
Item 4–Socio-professional status					0.346
*Manager*	31	58.49%	53	52.48%	
*Employee*	17	32.08%	24	23.76%	
*Student*	1	1.89%	3	2.97%	
*Retired*	1	1.89%	9	8.91%	
*Unemployed*	2	3.77%	10	9.90%	
Item 5—Are you in a “stable” relationship (i.e., relationship for many years, civil union, marriage?) (yes)	27	50.94%	47	47.00%	0.319
Item 6—Do you use means of protection during your sexual intercourse (condoms)?					0.006
*Yes*	8	15.09%	30	29.70%	
*Sometimes*	30	56.60%	33	32.67%	
Item 7—Do you practice Chemsex? (yes)	5	9.43%	16	16.33%	0.230
Item 8—Have you had any STI (chlamydia, gonorrhea, syphilis) in the past 3 months (yes)	18	33.96%	16	16.16%	0.014
Item 9—Have you been vaccinated against COVID-19 (yes)	50	96.15%	99	98.02%	0.505
Item 10—Do you feel at risk of being infected by MPXV? (yes)	40	75.47%	45	44.55%	<0.001
Item 11 Have you ever refused a vaccine because you consider it useless or dangerous.? (yes)	8	15.09%	12	11.88%	0.577
Item 12—Have you ever agreed to get vaccinated despite doubts about its effectiveness? (yes)	33	62.26%	63	62.38%	0.989
Item 13—As an adult, have you ever refused vaccination for reasons other than illness or allergy? (yes)	3	5.66%	11	10.89%	0.266
Item 14—People are getting more vaccines than needed? (yes)	8	15.09%	19	19.00%	0.543
Item 15—Vaccines are important to me to stay healthy. (yes)	49	92.45%	95	95.00%	0.531
Item 16—The proposed human smallpox vaccine is important in reducing the spread of the outbreak. (yes)	45	84.91%	90	89.11%	0.737
Item 17—The human smallpox vaccine should be compulsory for people at risk. (yes)	31	58.49%	56	55.45%	0.474
Item 18—Am-I likely to be more vulnerable to MPXV as a chronically ill patient? (yes)	-	-	57	60.64%	-
Item 19—Vaccination against MPXV is important for me as a patient with chronic disease. (yes)	-	-	67	71.28%	-
Item 20—I am concerned about serious side effects from the human smallpox vaccine. (yes)	17	32.08%	25	24.75%	0.198
Item 21—I need more information on the human smallpox vaccine than is given to the public now. (yes)	26	49.06%	49	48.51%	0.884
Item 22—I trust information I receive about the human smallpox vaccine from my doctor(s). (yes)	43	81.13%	85	85.00%	0.819
Item 23—Participant’s experience with MPXV (I personally know someone who has had a MPXV infection. (yes)	11	20.75%	12	11.88%	0.320
Item 24—Participant’s experience with MPXV (I was a contact case for the MPXV. (yes)	4	7.69%	2	1.98%	0.189
Item 25—Number of different sexual partners in the last month (mean/SD)	5.3	4.7	2.9	4.4	0.006
Item 26—Number of different sexual partners in the last three months (mean/SD)	11.8	12.2	5.5	8.3	0.002
Item 27—Duration of HIV (years) (mean/SD)	.	.	16.5	9.5	-

**Table 2:** Survey responses among the smallpox vaccine acceptance and hesitant groups in PrEP and HIV patients.

	PrEP Patients					HIV Patients				
	Vaccine Hesitant Group		Vaccine Acceptance Group		*p* Value	Vaccine Hesitant Group		Vaccine Acceptance Group		*p* Value
Item 1—Do You Want to Get Vaccinated against MPXV?	*n* = 11	20.7%	*n* = 42	79.3%		*n* = 41	40.2%	*n* = 61	59.8%	0.013
Item 2—Age					0.834					0.345
*18–29 years*	1	9.09%	4	9.52%		1	2.44%	3	4.92%	
*30–39 years*	5	45.45%	19	45.24%		2	4.88%	11	18.03%	
*40–49 years*	2	18.18%	12	28.57%		13	31.71%	16	26.23%	
*50–59 years*	3	27.27%	7	16.67%		16	39.02%	19	31.15%	
*Over 60 years of age*	0	0.00%	0	0.00%		9	21.95%	12	19.67%	
Item 3—Area of origin					0.122					0.482
*Africa*	0	0.00%	3	7.14%		3	7.32%	5	8.33%	
*America*	0	0.00%	2	4.76%		0	0.00%	3	5.00%	
*Asia/Oceania*	2	18.18%	2	4.76%		1	2.44%	3	5.00%	
Europe	8	72.73%	35	83.33%		37	90.24%	48	80.00%	
Item 4—Socio-professional status					0.409					0.609
*Manager*	6	54.55%	25	59.52%		23	57.50%	30	49.18%	
*Employee*	3	27.27%	14	33.33%		11	27.50%	13	21.31%	
*Student*	1	9.09%	0	0.00%		1	2.50%	2	3.28%	
*Retired*	0	0.00%	1	2.38%		2	5.00%	7	11.48%	
*Unemployed*	1	9.09%	1	2.38%		2	5.00%	8	13.11%	
Item 5—Are you in a “stable” relationship (i.e., a relationship for many years, civil union, marriage?) (yes)	6	54.55%	21	50.00%	0.788	15	38.46%	32	52.46%	0.346
Item 6—Do you use means of protection during your sexual intercourse (condoms)?					0.701					0.662
*Yes*	2	18.18%	6	14.29%		13	32.50%	17	27.87%	
*Sometimes*	7	63.64%	23	54.76%		11	27.50%	22	36.07%	
Item 7—Do you practice Chemsex? (yes)	0	0.00%	5	11.90%	0.229	6	15.00%	10	17.24%	0.768
Item 8—Have you had any STIs (chlamydia, gonorrhea, syphilis) in the past 3 months (yes)	3	27.27%	15	35.71%	0.598	5	12.50%	11	18.64%	0.415
Item 9—Have you been vaccinated against COVID-19 (yes)	9	81.82%	41	100.00%	0.006	38	95.00%	61	100.00%	0.078
Item 10—Do you feel at risk of being infected by MPXV? (yes)	5	45.45%	35	83.33%	0.032	3	7.50%	42	68.85%	<0.001
Item 11 Have you ever refused a vaccine because you considered it useless or dangerous? (yes)	3	27.27%	5	11.90%	0.205	10	25.00%	2	3.28%	0.001
Item 12—Have you ever agreed to get vaccinated despite doubts about its effectiveness? (yes)	6	54.55%	27	64.29%	0.553	22	55.00%	41	67.21%	0.215
Item 13—As an adult, have you ever refused vaccination for reasons other than illness or allergy? (yes)	3	27.27%	0	0.00%	<0.001	9	22.50%	2	3.28%	0.002
Item 14—People are getting more vaccines than needed. (yes)	5	45.45%	3	7.14%	0.002	13	33.33%	6	9.84%	0.003
Item 15—Vaccines are important to me to stay healthy. (yes)	9	81.82%	40	95.24%	0.133	35	87.50%	60	100.00%	0.005
Item 16—The proposed human smallpox vaccine is important in reducing the spread of the outbreak. (yes)	5	45.45%	40	95.24%	<0.001	31	77.50%	59	96.72%	0.009
Item 17—The human smallpox vaccine should be compulsory for people at risk. (yes)	4	36.36%	27	64.29%	0.239	12	30.00%	44	72.13%	<0.001
Item 18—I’m likely to be more vulnerable to MPXV as a chronically ill patient. (yes)	-	-	-	-	-	21	56.76%	36	63.16%	0.482
Item 19—Vaccination against MPXV is important for me as a patient with chronic disease. (yes)	-	-	-	-	-	15	40.54%	52	91.23%	<0.001
Item 20—I’m concerned about serious side effects from the human smallpox vaccine. (yes)	6	54.55%	11	26.19%	0.049	12	30.00%	13	21.31%	0.548
Item 21—I need more information on the human smallpox vaccine than is given to the public now. (yes)	9	81.82%	17	40.48%	0.047	21	52.50%	28	45.90%	0.195
Item 22—I trust information I receive about the human smallpox vaccine from my doctor(s). (yes)	8	72.73%	35	83.33%	0.536	32	80.00%	53	88.33%	0.215
Item 23—Participant’s experience with MPXV (I personally know someone who has had a MPXV infection. (yes)	1	9.09%	10	23.81%	0.392	2	5.00%	10	16.39%	0.199
Item 24—Participant’s experience with MPXV (I was a contact case for the MPXV. (yes)	1	9.09%	3	7.32%	0.803	0	0.00%	2	3.28%	0.005
Item 25—Number of different sexual partners in the last month (mean/SD)	2.8	2.3	6.0	4.9	0.019	1.1	1.6	3.9	5.2	0.002
Item 26—Number of different sexual partners in the last three months (mean/SD)	7.8	7.7	12.9	13.1	0.149	1.8	2.6	7.9	9.7	0.001
Item 27—Duration of HIV (years) (mean/SD)	.	.	.	.		17.8	9.6	15.7	9.4	0.315

**Table 3:** Multiple logistic regression for determinants of smallpox vaccine acceptance.

PrEP Patients			
Parameters	OR	IC 95%	*p* Value
Item 14—Are people getting more vaccines than needed? (yes)	0.10	[0.02–0.60]	0.012
Item 25—Number of different sexual partners in the last month (per units)	1.35	[1.04–1.93]	0.034
HIV patients			
Parameters	OR	IC 95%	*p* value
Item 10—Do you feel at risk of being infected by MPXV? (yes)	19.45	[6.14–33.48]	<0.001
Item 17—The human smallpox vaccine should be compulsory for people at risk? (yes)	15.58	[4.41–25.07]	<0.001
Item 25—Number of different sexual partners in the last month (per unit)	1.62	[1.08–2.49]	0.018

## Data Availability

The data that support the findings of this study are available on request from the corresponding author. The data are not publicly available due to privacy or ethical restrictions.

## Supplementary Materials

The following information can be download at: https://www.mdpi.com/article/10.3390/vaccines10101629/s1, Supplementary file: questionnaire for MPXV vaccination.
