# Supplementary material for: Monkeypox Vaccine Hesitancy in French Men Having Sex with Men with PrEP or Living with HIV in France
Source: Vaccines (Basel). 2022 Sep 28;10(10):1629. doi: 10.3390/vaccines10101629 (PMC9609833; doi:10.3390/vaccines10101629)
Supplement: Supplementary file 1 [file vaccines-10-01629-s001.zip › vaccines-1930466-supplementary.pdf]

Supplementary file: questionnaire for MPXV vaccination

Item 1 - Do you want to get vaccinated against MPXV?	Yes/nor for the moment/no
Item 2 - Age	*18-29 years / 30-39 years / 40-49 years / 50-59 years / more than 60 years old*
Item 3 – Area of Origin	Africa / America / Asia-Oceania / Europe
Item 4 - Socio-professional status	Manager / Employee / Student / Retired / Unemployed
Item 5 - Are you in a “stable” relationship (i.e., relationship for many years, civil union, marriage?)	Yes/no
Item 6 - Do you use means of protection during your sexual intercourse (condoms)?	Yes/Sometimes/no
Item 7 - Do you practice Chemsex?	Yes/no
Item 8 - Have you had any STI (chlamydia, gonorrhea, syphilis) in the past 3 months	Yes/no
Item 9 - Have you been vaccinated against COVID-19	Yes/no
Item 10 - Do you feel at risk of being infected by MPXV?	Yes/no
Item 11 Have you ever refused a vaccine because you consider it useless or dangerous.?	Yes/no
Item 12 - Have you ever agreed to get vaccinated despite doubts about its effectiveness?	Yes/no
Item 13 - As an adult, have you ever refused vaccination for reasons other than illness or allergy?	Yes/no
Item 14 - People are getting more vaccines than needed?	Yes/no
Item 15 - Vaccines are important to me to stay healthy.	Yes/no
Item 16 - The proposed human smallpox vaccine is important in reducing the spread of the outbreak.	Yes/no
Item 17 - The human smallpox vaccine should be compulsory for people at risk.	Yes/no
Item 18 – Am-I likely to be more vulnerable to MPXV as a chronically ill patient?	Yes/no
Item 19 - Vaccination against MPXV is important for me as a patient with chronic disease.	Yes/no
Item 20 – I am concerned about serious side effects from the human smallpox vaccine.	Yes/no
Item 21 - I need more information on the human smallpox vaccine than is given to the public now.	Yes/no
Item 22 - I trust information I receive about the human smallpox vaccine from my doctor(s).	Yes/no
Item 23 - Participant’s experience with MPXV (I personally know someone who has had a MPXV infection.	Yes/no
Item 24 - Participant’s experience with MPXV (I was a contact case for the MPXV.	Yes/no
Item 25 - Number of different sexual partners in the last month	Number 0 to 100
Item 26 - Number of different sexual partners in the last three months	Number 0 to 100
Item 27 - Duration of HIV (years)	Number 0 to 100
